# Placental dysfunction screening and perinatal loss

**DOI:** 10.1111/1471-0528.17197

**Published:** 2022-05-06

**Authors:** Becky Liu, Alexander Frick, Amar Bhide, Basky Thilaganathan

**Affiliations:** ^1^ Fetal Medicine Unit St George's University Hospitals NHS Foundation Trust London UK; ^2^ Vascular Biology Research Centre, Molecular and Clinical Sciences Research Institute St George's University of London London UK; ^3^ Tommy's National Centre for Maternity Improvement Royal College of Obstetrics and Gynaecology London UK

We thank these eminent academics for their interest in our work and for acknowledging the importance of stark ethnic health inequalities in maternity outcomes in the UK.^1^ We welcome open and balanced academic discourse but were disappointed to see the term ‘racial inequalities’ in their communication to the Journal. Race is perceived as inherent in biology, inherited across generations and therefore potentially unmodifiable. In contrast, ethnicity is understood as something we acquire based on factors like where we live, or the culture and interaction we share with others and as such, may be modifiable. The importance of this distinction was recently highlighted in the ethnic health inequalities report from the NHS Race and Health Observatory (NHS RHO).^2^ The link between race, ethnicity and health is complex, with black and minority ethnic groups paradoxically comparing favourably with white groups for some measures of health. What is evident is that black and minority ethnic groups are disproportionately affected by socio‐economic deprivation—a key determinant of health status. This may seem a trivial point, but this type of unconscious bias may lead to a lack of understanding of the causality (and solutions) to the health inequalities that we seek to address.

The pregnancy risk assessment prediction model used in our study was developed by the Fetal Medicine Foundation (FMF) and then externally validated in a large head‐to‐head comparison with the NICE risk assessment checklist in current use, in a study funded by the NIHR.^3^ The FMF then conducted a randomised trial to demonstrate the efficacy of a screening programme using the risk prediction model, followed by targeted aspirin prophylaxis and serial ultrasound scans; this significantly reduced the prevalence of preterm pre‐eclampsia.^3^ We implemented the protocol from this trial into a routine NHS setting and—using interrupted time series (ITS) analysis—demonstrated its effectiveness, with relative effect reductions in preterm pre‐eclampsia by 80% and term small‐for‐gestational age birth (SGA) birth by 45%.^3^, ^4^ In the strengths and limitations section of our manuscript we state the reasons why an ITS analysis would have been optimal, but that the total number of index events in the minimum required time period epochs before and after the intervention precluded this approach.

The correspondents raise the important point that the prediction model has not been investigated for its impact on perinatal death, and here the authors overlook two important issues. First, the use of a risk prediction model in isolation is very unlikely to change outcome in the absence of effective interventions. Secondly, pre‐eclampsia, SGA birth and stillbirth are outcomes of the overarching disorder of placental dysfunction. SGA and hypertensive disorders of pregnancy alone contribute approximately 30% of the population attributable risk to stillbirth as well as being major causes of preterm birth predisposing to neonatal death.^5^ Our previous publications on the effect of the screening programme on identifying reducing preterm pre‐eclampsia and term SGA birth is consistent with the finding in the current study of a much greater 72% reduction in PND associated with both of these conditions, compared with a 37% reduction in the overall population.^4^, ^6^ Pre‐eclampsia and SGA birth are more common in black and ethnic minority women, explaining the very specific finding of a three‐fold reduction in PND in this group of women compared with white women, in whom the PND rate was unchanged following the implementation of the screening programme. The correspondents also suggest that variable dose of aspirin in the before and after arms of the study may have biased results. To the best of our knowledge there are no studies that demonstrate efficacy of aspirin prophylaxis of any dose in preventing perinatal death, and such bias is therefore unlikely. We do not claim that implementation of the screening test alone accounted for the reduction in perinatal mortality—rather we made clear our hypothesis, namely, that the programme of interventions may have had a beneficial impact.

We agree with the authors that post‐hoc analysis when performed may be unreliable, but this does not translate as ‘all post‐hoc analyses are unreliable’. This is also true of secondary analyses conducted from randomised controlled trials (RCTs) and we are certain that the authors would not claim that all such analyses should be disregarded. We are unsure what groups the correspondents are referring to when they state that we relied on overlapping confidence intervals and non‐significant *P*‐values between study groups. We re‐iterate that in the (pre‐intervention) NICE cohort, the perinatal death rate was significantly higher in non‐white than white women (7.95 versus 2.63/1000 births, odds ratio [OR] 3.035, 95% confidence interval [CI] 1.551–5.941). Following the introduction of FMF screening (post‐intervention), the perinatal death rate in non‐white women was no longer significantly different from white women (3.22 vs. 2.55/1000 births, OR 1.261, 95% CI 0.641–2.483). Finally, they state that they did not find any subgroup effect in their test for interaction in the reported data. Presumably, they tested the difference between perinatal death rates between the NICE and FMF screened groups, adding ethnicity as an interaction term. Not finding a significant effect for this particular subgroup interaction test does not negate our study findings—as the authors themselves state their letter, is ‘a classic case of misinterpreting absence of evidence as evidence of absence’.

The problem of ethnic health inequality in maternity care and pregnancy outcomes has been evident for several decades.^7^ The COVID‐19 pandemic has brought such inequity to the fore and a plan of action in this area was proposed this week in the Government’s response to the Commission on Race and Ethnic Disparities report.^8^ The arbitrary use of screening thresholds in risk assessment has put the pursuit of perfection in the way of progress, leaving maternity services reliant on a checklist‐based approach developed 60 years ago. We do, however, acknowledge and understand the complex service/resource balance highlighted by Dr Ash Paul. The RCOG and RCM— funded by Tommy’s Charity—co‐developed the Tommy’s National Centre for Maternity Improvement (NCfMI), which is championing the use of digital health technology to produce personalised risk assessment in a collaborative and focused initiative to improve ethnic health inequalities.^9^ The NCfMI is actively seeking support, independent of current NHS funding, to undertake a study involving 20–30 hospitals. We look forward to working closely with healthcare practitioners, academics and policy makers in tackling the unnecessary and unwanted problem of ethnic and social disparity in maternal healthcare.

## DISCLOSURE OF INTERESTS

None declared. Completed disclosure of interest forms are available to view online as supporting information.

## AUTHOR CONTRIBUTIONS

All authors contributed to the writing of this correspondence.

## Funding information

Dr Becky Liu’s post was funded by an unrestricted grant from Biorithm Pte Ltd. The funders were not involved in the drafting of this correspondence.

## Supporting information


ICMJE

ICMJE

ICMJE

ICMJE

## Data Availability

Not applicable.

## References

[1] Liu B , Nadeem U , Frick A , Alakaloko M , Bhide A , Thilaganathan B . Reducing health inequality in black, Asian and other minority ethnic pregnant women: impact of first trimester combined screening for placental dysfunction on perinatal mortality. BJOG. 2022. 10.1111/1471-0528.17109 PMC9544950 35104381

[2] https://www.nhsrho.org/publications/ethnic‐health‐inequalities‐and‐the‐nhs/. [cited 2022 Apr].

[3] Chaemsaithong P , Sahota DS , Poon LC . First trimester preeclampsia screening and prediction. Am J Obstet Gynecol. 2022;226:S1071–S1097.e2. 10.1016/j.ajog.2020.07.020 32682859

[4] Guy GP , Leslie K , Diaz Gomez D , Forenc K , Buck E , Khalil A , et al. Implementation of routine first trimester combined screening for pre‐eclampsia: a clinical effectiveness study. BJOG. 2021;128(2):149–56. 32613730 10.1111/1471-0528.16361

[5] Cousens S , Blencowe H , Stanton C , Chou D , Ahmed S , Steinhardt L , et al. National, regional, and worldwide estimates of stillbirth rates in 2009 with trends since 1995: a systematic analysis. Lancet. 2011;377(9774):1319–30. 21496917 10.1016/S0140-6736(10)62310-0

[6] Guy GP , Leslie K , Diaz Gomez D , Forenc K , Buck E , Bhide A , et al. Effect of routine first‐trimester combined screening for pre‐eclampsia on small‐for‐gestational‐age birth: secondary interrupted time series analysis. Ultrasound Obstet Gynecol. 2022;59(1):55–60. 34319638 10.1002/uog.23741

[7] Muglu J , Rather H , Arroyo‐Manzano D , Bhattacharya S , Balchin I , Khalil A , et al. Risks of stillbirth and neonatal death with advancing gestation at term: a systematic review and meta‐analysis of cohort studies of 15 million pregnancies. PLoS Med. 2019;16(7):e1002838. 31265456 10.1371/journal.pmed.1002838 PMC6605635

[8] https://www.gov.uk/government/publications/inclusive‐britain‐action‐plan‐government‐response‐to‐the‐commission‐on‐race‐and‐ethnic‐disparities/inclusive‐britain‐government‐response‐to‐the‐commission‐on‐race‐and‐ethnic‐disparities. [cited 2022 Apr].

[9] https://www.rcog.org.uk/about‐us/groups‐and‐societies/the‐rcog‐centre‐for‐quality‐improvement‐and‐clinical‐audit/tommys‐national‐centre‐for‐maternity‐improvement/. [cited 2022 Apr].

